# Community health workers and mHealth systems for hearing screening in rural Nicaraguan schoolchildren

**DOI:** 10.7189/jogh.12.04060

**Published:** 2022-08-09

**Authors:** James E Saunders, Sarah Bessen, Isabelle Magro, Devin Cowan, Marvin Gonzalez Quiroz, Karen Mojica-Alvarez, Donoso Penalba, Catherine Reike, Christopher E Niemczak, Abigail Fellows, Jay C Buckey

**Affiliations:** 1 Department of Surgery Dartmouth-Hitchcock Medical Center, Lebanon, New Hampshire; 2 Dartmouth-Hitchcock Medical Center, Lebanon, New Hampshire, USA; 3 Geisel School of Medicine at Dartmouth, Hanover, New Hampshire, USA; 4 Research Centre on Health, Work and Environment (CISTA) at National Autonomous University of Nicaragua, Leon (UNAN-Leon), Leon, Nicaragua; 5 Centre for Nephrology, University College London, London, UK; 6 Medical Director, Mayflower Medical Outreach, Managua, Nicaragua; 7 Department of Public Health at National Autonomous University of Nicaragua, Leon (UNAN-Leon), Leon, Nicaragua.; 8 Space Medicine Innovations Laboratory, Geisel School of Medicine at Dartmouth, Lebanon, New Hampshire, USA; 9 Department of Medicine Dartmouth-Hitchcock Medical Center, Lebanon, New Hampshire, USA

## Abstract

**Background:**

We aimed to investigate the effectiveness of using minimally trained community health workers (CHW) to screen schoolchildren in rural Nicaragua for hearing loss using a tablet-based audiometric system integrated with asynchronous telehealth evaluations and mobile health (mHealth) appointment reminders.

**Methods:**

A population-based survey was conducted using community health workers (CHWs) to perform tablet-based audiometry, asynchronous telehealth evaluations, and mHealth reminders to screen 3398 school children (7-9 years of age) in 92 rural Nicaraguan communities. The accuracy of screening, test duration, testing efficiency, telehealth data validity, and compliance with recommended clinic visits were analyzed.

**Results:**

Minimally trained CHWs successfully screened children within remote rural schools with automated audiometry (test duration = 5.8 minutes) followed by manual audiometry if needed (test duration = 4.3 minutes) with an estimated manual audiometry validity of 98.5% based on a review of convergence patterns. For children who were referred based on audiometry, the otoscopy and tympanometry obtained during telehealth evaluations were high quality (as reviewed by 3 experts) in 44.6% and 80.1% of ears, respectively. A combination of automated short message service (SMS) text messages and voice reminders resulted in a follow-up compliance of 75.2%. No families responded to SMS messages alone.

**Conclusions:**

Tablet-based hearing screening administered by minimally trained CHWs is feasible and effective in low- and middle-income countries. Manual audiometry was as efficient as automated audiometry in this setting. The physical exam tasks of otoscopy and tympanometry require additional training. Mobile phone messages improve compliance for confirmatory audiometry, but the utility of SMS messaging alone is unclear in this population.

Pediatric hearing loss is a major public health problem that impairs the educational and economic outcomes of children. An estimated 34 million children have disabling hearing loss (>30 dB) [1-4] and another 29 million children have mild hearing loss (>20 dB) that may be educationally significant [5]. According to the World Health Organization, roughly 60% of the burden of pediatric hearing loss is avoidable through primary, secondary, and tertiary prevention measures [4.] However, most of these children live in low- and middle-income countries (LMICs) where hearing loss often goes unrecognized and untreated [1-4]. Thus, systematic public health approaches are needed to identify and prevent hearing loss in LMICs. Such efforts also support the 4th Sustainable Goal of the United Nations: “Universal Primary Education” to “ensure inclusive and equitable quality education and promote lifelong opportunities for all” [6].

When combined with effective intervention, school-based hearing screening is an effective public health strategy to prevent hearing loss disability. [7-10]. However, these services are rarely available in LMICs [11-13]. Many Latin American countries, including Nicaragua, do not provide routine hearing screening in public schools, despite evidence that pediatric hearing loss is common in these populations [14-17]. Screening protocols in these limited resource environments must balance cost with test accuracy, as inaccurate testing may create false-positive results which lead to unnecessary referrals that consume limited medical resources [17,18]. The costs of these programs in LMICs might be reduced by shifting screening tasks to community health workers (CHWs) with targeted training and by utilizing relatively low-cost tablet-based audiometry [19-23]. However, few studies have investigated the validity and effectiveness of this approach. A small-scale pilot study by our group employed a tablet-based audiometer paired with noise attenuating wireless headphones and a two-step protocol (automated audiometry followed by confirmatory manual audiometry for initial referrals) among Nicaraguan school children. It showed accurate audiometric results with a sensitivity of 100% and specificity of 99% for detecting hearing loss [24].

Effective follow-up after referral can be challenging for children in LMICs due to poverty, poor access to transportation, misinformation, cultural bias, or distance to the clinic [25-27]. Our previous anecdotal experience with hearing screening in Nicaraguan schools has shown that <10% of referred children complete their recommended evaluations, even when written recommendations are provided to teachers and families. Mobile health (mHealth) systems have been proposed to improve patient compliance, by tracking screening test results and facilitating communication between patients and providers [28-33]. The effectiveness of mHealth interventions for school-based hearing screening, however, has not been studied.

We have conducted a comprehensive analysis of a large-scale, population-based survey of school hearing screening in a remote, mountainous region of rural Nicaragua. This project employed minimally trained CHWs to perform tablet-based audiometry and asynchronous telehealth evaluations, which were then linked to a mHealth system that incorporated automated short message service (SMS) messaging to improve follow-up compliance. The focus of this report is to: 1) evaluate the effectiveness of minimally trained CHW to detect hearing loss reliably and perform the necessary asynchronous telehealth examinations (including otoscopy and tympanometry), and 2) examine the capacity of the mHealth system to improve compliance with recommended referrals (ie, audiology and otolaryngology). Other aspects of this work including automated audiometry reliability, specific audiometry results, and clinical findings are presented elsewhere [34,35].

## METHODS

### Participants

The Ministry of Education provided a registry of students in schools in the Department of Jinotega, Nicaragua. A proportional, stratified sample of children aged 7-9 years was recruited from the eight municipalities of Jinotega, representing a 10% cross-sectional sample of all children in this age range living in this region, proportionally distributed based on the school children population in each municipality. The Jinotega Ministry of Education coordinated the dates and sites of testing between October 2018 and November 2019. Families and teachers were notified of the screening event one month prior to testing and given the opportunity to opt out of the study.

### Personnel and training

CHWs from the region with no prior audiological training or experience with hearing screening were recruited to perform the screening exams. No health care experience was required. A skills assessment relevant to the project was used to select three CHWs (two recently graduated nurses and one community organizer) out of 22 applicants.

The CHWs completed video training modules that were developed in Spanish specifically for this project. These video training modules detailed automated and manual audiometry techniques using the open-source hearing assessment software TabSINT, the testing protocol, noise measurement, video otoscopy and tympanometry. The entire length of these video modules was 22 minutes. The modules were available on the testing tablets for CHW review at any time during the project. Following completion of these modules, the technicians attended two in-person training sessions (eight hours each) led by a Nicaraguan otolaryngologist (KM), which included additional presentations (approximately two hours) on the study protocol and the Hughson-Westlake audiometric methods to determine the hearing threshold, otoscopy, and tympanometry. The remainder of this two-day training was spent with hands-on training with the equipment performing audiometry, otoscopy, and tympanometry on each other and four adult volunteers. This level of technical training for the CHWs in this study is comparable to a Level 2 CHW (paraprofessional) as described by Olaniran et al. [36].

### Equipment

Tablet-based hearing threshold testing was performed using a tablet-based system (WAHTS, Creare, LLC, Hanover, NH) which incorporates a calibrated, wireless audiometric speaker into highly noise-attenuating, over-the-ear headphones that are connected to a commercially available tablet using Bluetooth wireless technology [37,38]. The sound attenuation of the WAHTS headphones for the frequencies included in this study is 36-42 dB of attenuation, comparable to a single-walled sound booth. Annual calibration is recommended for this system and calibration was confirmed prior to the beginning of this study. Due to pandemic travel restrictions, calibration could not be rechecked until two years after the completion of the study. At that time, all included frequencies (1, 2, and 4 kHz) on the three WAHTS headphones used in this study were still in calibration.

**Figure 1:**
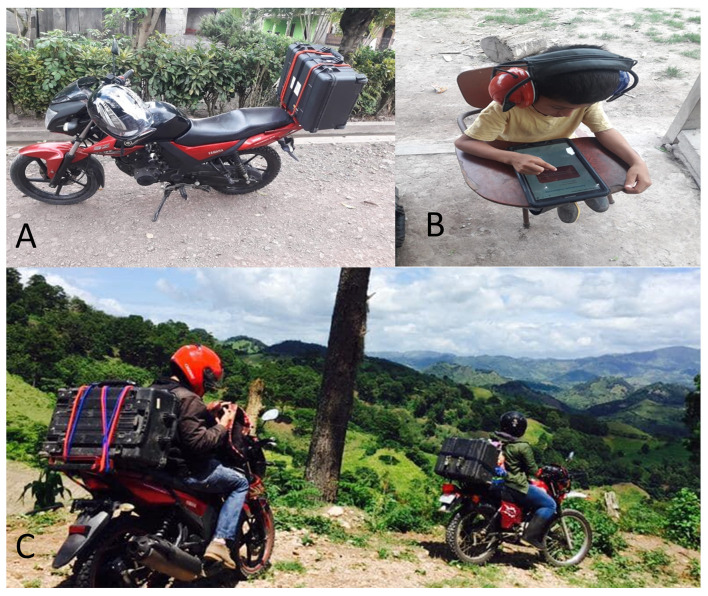
Transportation of portable audiometric equipment to remote testing sites: A) Complete tablet-based audiometric system with WAHTS mounted on motorcycle for transport. B) Student completing tablet-based automated audiometry with WAHTS headset. C) Community Health Workers en route to remote mountainous study site.

The automated and manual testing protocols were created using open-source TabSINT software (Creare, LLC, Hanover, NH) [39]. All necessary testing equipment (WAHTS headset, tablet, sound level meter, digital otoscope, and tympanometry) were packaged in a hard case mounted on a motorcycle rack to visit these remote communities (**Figure 1**). All subject data were directly entered into a tablet-based research database (REDCap). TabSINT audiometric data and REDCap data were synced by using a unique Q/R code identifier. Once a cellular internet connection was available, data were uploaded into a REDCap-based mHealth hearing management system (mHMS).

### School hearing screening protocol

The CHWs travelled to primary schools throughout the Department of Jinotega by motorcycle to perform hearing testing on all children within the designated age range. In each community, school officials or community leaders were asked to complete a survey covering local socioeconomic conditions and telecommunication capacity (eg, internet access and mobile phone coverage in the schools). A pre-test ambient noise measurement (PCE-353, PCE Instruments, Jupiter, Florida) of <55dBA was required prior to automated and manual threshold testing at 1, 2, and 4 kHz.

### Automated audiometry

Each child viewed an instructional video and completed a practice trial of automated audiometry before testing. Children were asked to press a large red button on the tablet when they heard a tone. Automated WAHTS testing consisted of modified Hughson-Westlake audiometry to determine audiometric thresholds (rather than the presentation of suprathreshold stimuli) for 1, 2, and 4 kHz. The details of the test parameters are described elsewhere [34]. If the CHW noticed that the child was having difficulty with automated audiometry (eg, inattentiveness or pressing the button at regular or erratic intervals), they could identify the child as having “difficulty with testing”, abort the automated test, and refer for manual audiometry [33]. Referrals to manual audiometry were initiated for thresholds >25 dBHL or for invalid thresholds (a failure to converge or a threshold outside the measurable range (<-10 dB or >80 dB)).

### Manual audiometry

Tablet-based manual audiometry used a modified Hughson-Westlake audiometry with children facing away from the CHW and raising their hand if they heard a tone. The CHW controlled the tone presentation level and interval. Pure tone audiometric thresholds were determined at 1, 2, and 4 kHz. The TabSINT system recorded all presentation levels and thresholds for later analysis.

### Asynchronous Telehealth Evaluation

Students who were referred on manual audiometry were subsequently referred for detailed telehealth evaluation (TE) performed by the CHW in the family home on the same day of screening. Parental mobile phone number and GPS location were recorded. Parents were interviewed in Spanish regarding risk factors for hearing loss including reports of ear pain or drainage, prior medical history, prenatal history, family history, and environmental exposures. Responses were entered into the tablet-based REDCap database. Technicians also performed digital otoscopy (Firefly DE500 Digital Video Otoscope, Firefly Global; Belmont, Massachusetts) and tympanometry (MT10 Handheld Impedance Tympanometer, Interacoustics, Middelfart, Denmark). A post-hoc blinded review of the otoscopy images was performed for image quality (based on a 3-point grading scale) and presence of cerumen impaction. Likewise, tympanometry data were reviewed for validity (analysis of ear canal volume and tympanogram type) and compared to the otoscopy images.

### mHealth platform and appointment reminders

The home-based evaluation and exam results (digital otoscopy images and tympanometry data) were uploaded to the REDCap-based mHMS and reviewed by an otolaryngologist (KM) who determined the need for and urgency of clinical referral. That decision was entered into the mHMS, which generated an email to the technicians who then coordinated an appointment for a follow-up at the Otolaryngology and Audiology Clinic at Victoria Motta Hospital in Jinotega (OAC-VMH). The appointment date and time were entered into the mHMS, which automatically interfaced with a US-based communications platform (Twilio). This platform generated automated text message reminders (in Spanish) for appointments to families and instructed the recipient to confirm or cancel the appointment. Text messages (SMS) were sent weekly until the subject was within seven days of the appointment date, at which point a text was sent daily until the appointment. Unfortunately, this initial protocol design did not account for the fact that the SMS messages were generated in the United States and that families would incur cellular charges to respond to these appointment reminders. This SMS text generating system is not available with local Nicaraguan phone numbers. No families responded to the SMS messages. SMS text message reminders continued to be sent, but the protocol was subsequently changed to stop asking families to respond to the SMS messages. If an appointment was missed, voice phone call appointment reminders were made in addition to SMS messages at weekly intervals.

Manual audiometry validity was determined by a review of sampled convergence patterns (presentation levels) and recorded thresholds. Mathematical criteria for valid threshold convergence patterns were developed. All presentations violating these mathematical criteria were reviewed by an audiologist (CR) and neurotologist (JS). In addition, a 10% sample of those meeting the criteria (829 convergences patterns) were reviewed to confirm the model’s validity. The estimated manual audiometry validity for each CHW was derived by summing the known invalid thresholds that failed to meet the mathematical criteria and the estimated percentage of invalid thresholds meeting the criteria. A bootstrapping model was used to determine the uncertainty of the manual audiometry validity measures. Manual audiometry validity was compared for each CHW using a χ^2^ analysis. Otoscopy image quality and cerumen impaction was independently reviewed by three reviewers, including one audiologist (CR) and two neurotologists (JS and SW), and a composite score was derived. Images in which the tympanic membrane was fully visible and in focus were rated as high-quality. Tympanometry validity was assessed based on abnormally low canal volumes, the presence or absence of cerumen, and the clinically derived tympanogram. CHW-specific data on otoscopy and tympanometry were not available. Right and left ears were compared for otoscopy and tympanometry.

### Ethics approval

The Committee for the Protection of Human Subjects at Dartmouth College, the Ministry of Education of the Department of Jinotega, and the Institutional Review Board of the National Autonomous University of Nicaragua, Leon and the Nicaraguan Ministry of Health approved the project. The rationale and description of the study design have been presented elsewhere [34,35].

## RESULTS

### Participant characteristics

**Figure 2:**
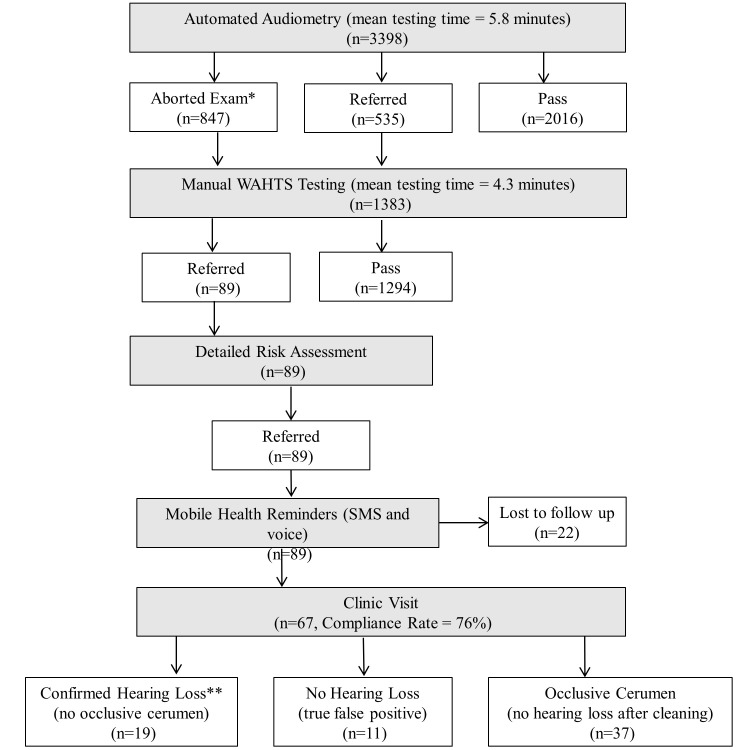
Flowchart of audiometric screening protocol with number of participants at each stage. *Aborted automated audiometric due to difficulty with testing or behavioral problems, **No detectable hearing loss at screened frequencies in school (1000, 2000, and 4000 Hz) – children with hearing loss outside of this range were not included in this result.

**Figure 3:**
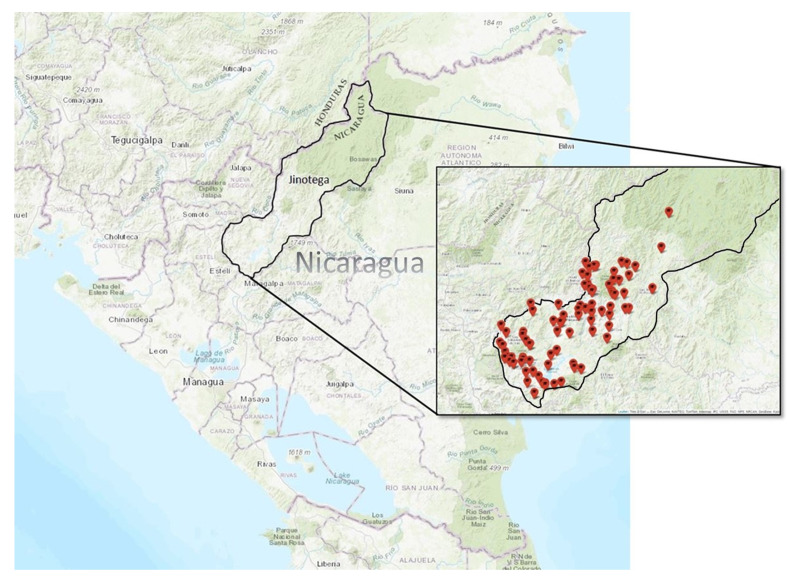
Geographic distribution of testing sites in the Department of Jinotega in northern Nicaragua.

Hearing testing occurred between October 22, 2018, and November 15, 2019. A total of 3398 children from 92 schools from municipalities in the Department of Jinotega were enrolled in the study. Only one family declined to participate. The demographic characteristics of the participants are presented in **Table 1**. The mean age of the participants was 7.96 years (range = 7-9). **Figure 2** illustrates a flowchart of the study protocol and the number of participants tested. The schools visited were widely distributed throughout the rural mountainous region of the Jinotega Department (**Figure 3**).

**Table 1:** Participant characteristics

	Total sample (n = 3398)	Referred for detailed telehealth evaluation (n = 89)
**Age (n, %)**		
7	1183 (34.8%)	42 (47.2%)
8	1145 (33.7%)	20 (22.4%)
9	1070 (31.5%)	27 (30.3%)
**Gender (n, %)**		
Female	1741 (51.2%)	43 (48.3%)
Male	1657 (48.8%)	46 (51.7%)
**Grade (n, %)**		
1	670 (19.7%)	22 (24.7%)
2	1198 (35.3%)	34 (38.2%)
3	990 (29.1%)	18 (20.2%)
4	490 (14.4%)	13 (14.6%)
5	48 (1.4%)	2 (2.2%)
6	2 (0.1%)	0 (0%)

### Testing parameters and automated audiometry

Selected details of the telecommunication survey from 89 communities (97%) representing 3016 children are shown in **Table 2**. Only 58 (65.2%) of study site schools had electricity. Internet access was rare with wireless (2.3%) or fixed internet (1.1%) available in the schools for only a small portion of study sites. In contrast, 79.8% of students attended schools with mobile phone coverage and survey respondents estimated that 78% of people living in those 89 communities have access to mobile phones. Five communities (5.6%) reported mobile phone access of less than 50%. Of the 3398 students who underwent at least one trial of automated audiometry, 1382 (40.7%) were referred to manual audiometry. The mean time to complete the first automated test was 5.8 minutes (SD = 2.1). There was no significant difference in automated referral rates for the three CHWs (*P* = 0.34).

**Table 2:** Socioeconomic and telehealth survey for 92 communities

Survey question	n (%)
Feel that child hearing loss is a problem in the community	26 (29.2%)
Personally know children with hearing loss	19 (21.3%)
% of population completed secondary education	20 (22.5%)
Average income<US$100 in past year	73 (82.0%)
100% of children receive all recommended vaccines	54 (60.7%)
Children receive hearing screening in schools	2 (2.2%)
Most families work with pesticides	84 (100.0%)
Most families have pesticides in the house	80 (98.8%)
Presence of a computer in this school/clinic (n, %)	13 (14.6%)
Presence of smart phones or tablets in this school/clinic (n, %)	10 (11.2%)
Presence of wireless Internet connection in the clinic/school (n, %)	2 (2.3%)
Access to Internet nearby (n, %)	3 (3.4%)

### Manual audiometry

Of the 1382 students who completed manual audiometry in the field, 89 (6.4%) were referred for a telehealth evaluation for an overall referral rate of 2.6% (89/3398). There was no significant difference in rates of referral for manual audiometry for the different CHWs (*P* = 0.539). The mean time to complete manual audiometry was 4.3 minutes (SD = 1.5 minutes).

To verify the accuracy of the mathematical criteria to detect invalid threshold convergence patterns, 10% (829/8283 thresholds) of manual audiometry convergence patterns meeting the established validity criteria were reviewed (two reviewers). 3/829 were considered invalid (0.25%). All the convergence patterns that failed to meet the mathematical criteria were reviewed. Of those, 94.8% (74/78) were considered invalid. Combining the estimated and documented invalid convergence patterns resulted in a mean overall manual audiometry validity of 98.5% for all CHWs with no significant difference between the CHWs (*P* = 1.00). To calculate the uncertainty in the audiometric validity we used a bootstrapping approach. From our analysis of a sample of the thresholds from the CHWs, we found that the percentage of invalid tests ranged from 0%-0.48%. To calculate the variability in the audiometric validity results a percentage invalid for tests meeting criteria was randomly selected from the range of 0%-0.48% and the percentage of invalid tests (ie, the sum of the confirmed and estimated invalid tests) out of the total tests was calculated 1000 times. The calculated mean accuracy using this bootstrap approach of multiple iterations was slightly higher at 98.8% ± 0.14% (range = 98.6-99.1).

### Asynchronous telehealth evaluation

A total of 89 children were referred for a detailed exam and risk assessment survey. Two families refused the assessment. Most families had access to more than one mobile device; however, eight families (8.9%) declined to provide a mobile phone number or did not have access to a mobile phone. No children had been previously tested for hearing loss.

Otoscopy was attempted in 100% of children and otoscopic images were obtained from both ears in 88/89 (98.9%) children. One otoscopic image was not obtained due to microtia and external auditory canal atresia of the right ear in one child. The ear-specific otoscopic image quality results (3 reviewers) are shown in **Table 3**. Image quality was considered excellent for only 79/178 images (44.6%) and poor for 8/178 images (4.5%) with no difference between the right and left ear image quality (*P* = 1.00).

**Table 3:** Telehealth evaluation findings (n = 89 children)

	Right ear, n = 88	Left ear, n = 89
**Otoscopy image quality**		
Excellent Quality, n (%)	39 (44.3)	40 (44.9)
Moderate Quality, n (%)	45 (51.1)	45 (50.6)
Poor Quality, n (%)	4 (4.5)	4 (4.5)
**Tympanometry**	n = 86	n = 87
Invalid Tympanometry (n = 126 ears)*, n (%)	14 (22.6)	11 (17.2)

*Tympanometry validity was only assessed for those children with clinic-based tympanometry results.

Tympanometry was performed in 86 (97%) of right ears and 87 (98%) of left ears. For the 65 children with available data from the clinic visits, the tympanogram subtypes in the field matched the clinic tympanograms in only 48 right ears (77.4%) and 53 left ears (82.8%). Altogether, 14 (22.6%) of right ear tympanograms and 11 (17.2%) of left ear tympanograms in the field were considered invalid. There was no significant difference in tympanogram validity between the left and right ears (*P* = 0.48). The validity of the tympanogram in the field was uncertain in four ears (three right and one left).

### Clinical assessment

89 participants were referred for clinic visits and 67 (75.2.%) completed the visit. Text message reminders were sent to 81 (92.0%) families. Within this group, a mean of 22.5 (range = 2-57) SMS messages and 3.8 (range = 3-5) live voice calls were performed. Seven families attended the clinic before the SMS was generated by the system. Families that completed their follow-up appointment had a shorter average travel time required to get to the audiology clinic (mean = 5.7 hours, SD = 2.9) compared to those who did not comply with the follow-up recommendations (mean = 8.2 hours, SD = 9.5), although this difference was not statistically significant (*P* = 0.4). Of the 67 children with confirmatory audiometric data, 13 (19%) were found to have normal hearing (false positive) after transient causes such as cerumen impaction were excluded. When those with manual audiometry referrals who failed to complete confirmatory audiometry were excluded, the calculated specificity of the screening system to detect any type of hearing loss (including cerumen impactions) was 99.6% and the positive predictive value was 77%. Further details of the clinic exam findings are reported elsewhere [35].

## DISCUSSION

Our study demonstrates that minimally trained CHWs can perform both automated and manual audiometry effectively using tablet-based audiometry and a noise-attenuating headset in rural Nicaraguan schools. This portable technology eliminates the need for a sound-proof booth and allows accurate screening in these challenging remote locations. Shifting tasks to CWHs and incorporating remote telehealth and mHealth technologies are crucial to providing these services due to limited professional resources in low-resource settings [36,40-44]. The CHWs employed in this study would be considered at a higher level of training (Level 2 paraprofessional) [36]. Nevertheless, audiological or medical training was not a pre-requisite for this project, and we found no differences in outcomes for the CHWs who did not have prior health care experience. Our results agree with published reports of task shifting in LMICs demonstrating referral rates that are comparable to hearing screening programs administered by otolaryngologists or audiologists [40-45]. Furthermore, families were generally receptive to CHWs providing these services. Only two families (2.2%) declined to have the CHWs visit their home, suggesting a high acceptance rate.

Automated audiometry has been proposed as one approach to facilitating screening in school children [46-51]. However, there are concerns regarding the accuracy and reliability of automated audiometry in young children [20]. Our previous experience with Nicaraguan children demonstrates a higher specificity when automated audiometry was coupled with manual audiometry [34]. We have now demonstrated that CHWs can perform manual audiometry effectively with high validity (98.5%) with shorter testing times than automated audiometry (4.28 minutes vs 5.80 minutes). Occasional invalid responses seen in the convergence pattern analysis for manual audiometry may be due to transient noise effects or erratic testing behaviour, as we have described in a previous report on automated audiometry in this population [34]. Importantly, both the automated and manual audiometry protocols in this study employed threshold audiometry to measure audiometric thresholds, not merely a response to suprathreshold stimuli. Also, despite the additional complexity for manual audiometry, CHWs performed remarkable well. These findings suggest manual audiometry by CHWs may be the preferred screening protocol in this population.

Smartphone-based digital otoscopy has been endorsed for use in telehealth exams by physicians and parents, but few studies have investigated this technology in CHW-based screening [52-56]. Our results reveal that digital otoscopy and tympanometry are the greatest challenges for CHWs, with either marginal quality or invalid data in 44.3% and 19.8%, respectively. These findings reflect the challenge of training for these “hands-on” exam skills. Other studies have addressed the importance of CHW training requirements for performing specific hearing-related tasks, but little consideration has been given to specific examination skills [42,44,51,57]. Our results suggest that audiometry requires less training than otoscopy and tympanometry. Cerumen impaction is common in LMIC schoolchildren and can produce unnecessary screening referrals, as well as reduce the quality of otoscopy and tympanometry data [58]. Additional training in cerumen management for CHWs evaluating children who referred on initial testing would also be valuable in this remote setting and should be considered for future screening programs.

Despite the remote testing locations and limited internet access, the CHWs uploaded data successfully from tablet-based audiometry and telehealth evaluations to the mHMS platform. Most, but not all, participating families had mobile phones and most communities had mobile phone signals at the school. With these mHealth reminders, the follow-up compliance rate was much higher (75.2%) in this study than in our previous study in the same region (<10%) and studies conducted in other LMICs (3%-27%) [59-62]. This is likely related to the mHMS tracking of follow-up appointments, multiple SMS reminders, and live voice calls to families [52,62]. The extent to which the mHealth SMS texting affected compliance in our study remains unclear. The reported effectiveness of SMS reminders in LMICs is controversial. A recent systematic review reported that only 50% of studies demonstrated improved compliance with SMS reminders [29]. Another systematic review reported an average 30% increase in compliance over baseline and concluded that voice calls were more effective than text messaging alone [30]. In our study, a combination of SMS and live voice calls was necessary in 99% of cases, suggesting SMS reminders alone may be insufficient. Furthermore, mobile phone access is not ubiquitous in these rural LMICs communities. Although most families had access to multiple devices, a small sub-set (8.9%) of our cohort did not have access to mobile technology.

These results support the argument that other factors not addressed by appointment reminders affect patient compliance in LMICs. Multiple cultural and practical barriers are known to reduce compliance rates after hearing screening (eg, lack of awareness, distance to the clinic, lack of funds for travel, or other indirect costs) [27,28,60]. The primary difficulty families face is long travel times to the clinic for both the compliant group (5.7 hours) and those who never came to the clinic (8.2 hours). These long distances and the multiple reminders required to achieve the 75.2% compliance rate in our study suggest that increasing management at the local level with synchronous telehealth and point-of-care cerumen management may reduce referrals and improve compliance, as well as enhance detection of other ear pathologies. Furthermore, this would allow for point-of-care evaluations for those children whose families do not have access to mobile technology. Future studies will focus on the relative cost-effectiveness of these approaches.

This study has certain limitations. We cannot fully assess the benefit of SMS messaging alone, as most families were contacted by both SMS and voice messages. Likewise, the study lacks a control group that did not receive appointment reminders. Our comparison compliance rate is based on our unpublished experience in the same region. We also lack CHW-specific data that would allow comparisons between CHWs on some specific tasks (eg, otoscopy and tympanometry). Finally, our results are specific to rural Nicaragua. Although this population shares many attributes common in LMIC populations (eg, wide geographic distribution, low population density, low socioeconomic status, and poor telecommunication access), our experience may not necessarily be translatable to other cultures or to more urban settings. Nonetheless, our results show that using CHWs and tablet-based audiometry improves access to school-based hearing screening in rural LMICs, has a low rate of unnecessary referrals, and increases patient compliance.

## CONCLUSIONS

Minimally trained CHWs were able to complete automated and manual tablet-based audiometry effectively on rural Nicaraguan schoolchildren and upload asynchronous telehealth data consistently from these remote encounters. The combination of an internet-based appointment tracking system and mobile communication (both SMS and voice) improved patient compliance with recommended clinic appointments. Competency in physical exam skills (otoscopy and tympanometry) requires additional training.
